# Automated numerical simulation of biological pattern formation based on visual feedback simulation framework

**DOI:** 10.1371/journal.pone.0172643

**Published:** 2017-02-22

**Authors:** Mingzhu Sun, Hui Xu, Xingjuan Zeng, Xin Zhao

**Affiliations:** 1 Institute of Robotics and Automatic Information Systems, Nankai University, Tianjin, China; 2 Tianjin Key Laboratory of Intelligent Robotics, Tianjin, China; Shanxi University, CHINA

## Abstract

There are various fantastic biological phenomena in biological pattern formation. Mathematical modeling using reaction-diffusion partial differential equation systems is employed to study the mechanism of pattern formation. However, model parameter selection is both difficult and time consuming. In this paper, a visual feedback simulation framework is proposed to calculate the parameters of a mathematical model automatically based on the basic principle of feedback control. In the simulation framework, the simulation results are visualized, and the image features are extracted as the system feedback. Then, the unknown model parameters are obtained by comparing the image features of the simulation image and the target biological pattern. Considering two typical applications, the visual feedback simulation framework is applied to fulfill pattern formation simulations for vascular mesenchymal cells and lung development. In the simulation framework, the spot, stripe, labyrinthine patterns of vascular mesenchymal cells, the normal branching pattern and the branching pattern lacking side branching for lung branching are obtained in a finite number of iterations. The simulation results indicate that it is easy to achieve the simulation targets, especially when the simulation patterns are sensitive to the model parameters. Moreover, this simulation framework can expand to other types of biological pattern formation.

## Introduction

Biological patterns with specificity and different functions are formed from single cells in the development of higher organisms. It is reported that this process is controlled by a complex network of biochemical reactions, which are under genetic control[1–3]. Research on the mechanism of biological pattern formation is one of the key problems in developmental biology. Although this problem has been investigated in the field of molecular biology[4, 5], biochemistry[6], mathematics[7–10], mechanics[11, 12], and epidemiology[13–16] for many years, it has long remained unclear.

In the study of biological pattern formation mechanisms, a prominent approach is the use of mathematical models to investigate the logic of patterning. The mathematical model, which is usually a partial differential equation (PDE) system, describes the reaction and diffusion of some chemicals, i.e., the so-called activator and inhibitor, and represents the biological pattern by the solution of the PDE system. Many of these models have been supported by biological experiments, such as the shells of molluscs[17], the skin of snake[18], the skin of marine angelfish[19, 20], and vascular mesenchymal cells (VMCs) self-organization[21, 22], the simulation results are similar to the biological phenomena that are observed in experiments.

In most cases, an analytical solution cannot be obtained in a PDE system. The numerical solution of a PDE system depends on the parameters of the equations, which represent the biological or chemical conditions in the biological pattern formation. Because there are usually many parameters in a PDE model, it is critical to select the appropriate values for the parameters after the model is determined. In previous studies, researchers needed to try large quantities of parameter combinations manually to obtain a satisfactory simulation result. Although the parameter scopes can be narrowed by mathematical analysis, it still takes a long time to complete the numerical simulation. For example, Eldar et al., in an investigation of drosophila embryonic patterning, tried a total of 66000 parameter combinations in a reaction-diffusion model and solved all of them numerically[23].

Some methods that estimated the parameters in pattern formation models were applied to the study of Drosophila melanogaster and Drosophila gap gene circuits [24, 25]. These methods accelerate the simulations by optimization algorithm, but we need to design new method for each model. In fact, the simulation result of the biological pattern formation is visualized as an image. We evaluate the mathematical model and the model parameters by comparing the simulation image to the image of the target biological pattern. A good simulation result has the same pattern topology and similar pattern features as the target pattern. Taking pattern formation by VMCs as an example[21], we first compare the pattern topology, such as spots, stripes or labyrinths, and we then compare the quantitative pattern features, such as the size of the spots or the quantity of the stripes. The most similar simulation result is selected last. The method of manual simulation guides us to achieve automated numerical simulation based on visualization and feature comparison.

In this paper, a visual feedback simulation framework is proposed by applying feedback control theory to calculate the unknown parameters of the mathematical model automatically. In the simulation framework, the mathematical model and the numerical solution are analogous to the controlled plant and the system output in control system. The pattern topology and quantitative pattern features of the visualized numerical solution are extracted as system feedback and compared to those of the target pattern (system input) to obtain the model parameters (control input). The simulation framework searches model parameters nonlinearly in high-dimensional parameter space, which is more efficient than manual searches.

Mesenchymal stem cells self-organization and organ branching are two types of typical biological pattern formation and have been investigated by many researchers through biological experiments or mathematical analysis[26, 27]. In this paper, we design and implement the visual feedback simulation framework and fulfill pattern formation simulations for VMCs and lung development as two typical applications. The simulation processes and results indicate that this simulation framework is effective and efficient, and it is easy to expand to other types of biological pattern formation.

## Methods

### Visual feedback simulation framework for pattern formation

**Fig 1:**
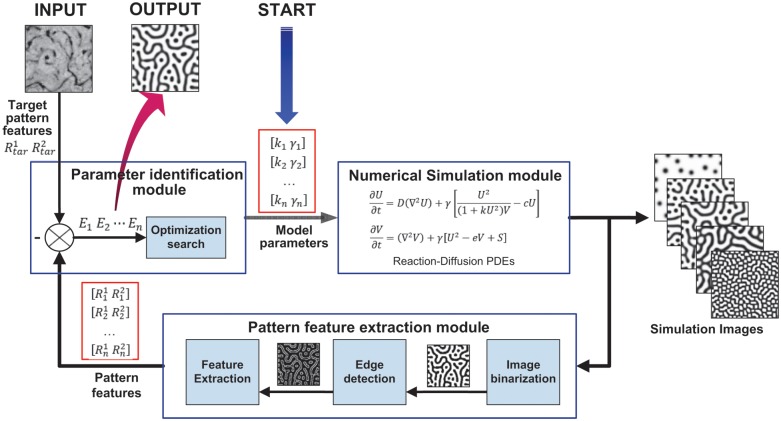
The structure and process of the visual feedback simulation framework. The simulation framework consists of three modules: the numerical simulation module, pattern feature extraction module and parameter identification module. Taking VMCs pattern formation simulation as an example, the visual feedback simulation framework calculates two unknown parameters *k*, *γ* of the mathematical model, according to the pattern feature differences between the target biological pattern and the simulation images. The numerical solution with the optimal evaluation value is outputted as the simulation result of the biological pattern.

The visual feedback simulation framework calculates the unknown parameters of the mathematical model, according to the visual differences between the target pattern and the simulation result, and then, it obtains a numerical solution of the model. As shown in Fig 1, the simulation framework consists of three modules:

- Numerical simulation module: This module is equivalent to the actuator in the feedback control system. In this module, the mathematical model with determined parameters is solved, and the numerical solution is saved as an image as the system output.
- Pattern feature extraction module: This module is equivalent to the sensor in the feedback control system. In this module, the pattern topology and quantitative pattern features of the simulation image are extracted as system feedback.
- Parameter identification module: This module is equivalent to the controller in the feedback control system. In this module, the pattern topology and quantitative pattern features of the simulation image are compared to those of the target pattern (system input) to evaluate the unknown model parameters. Then, groups of model parameters and their evaluations are utilized to calculate the new model parameters.

The visual feedback simulation framework is designed as a software framework. We choose the appropriate mathematical model according to the target biological pattern before running the program because different phenomena of biological pattern formation are modeled by different types of reaction-diffusion PDE systems. As a result, the simulation framework can be applied to various biological pattern formation simulations. In the simulation framework, we optimize the model parameters by using a differential evolution (DE) algorithm[28]. As a type of genetic algorithm, the DE algorithm is a simple and fast heuristic approach to obtaining a global optimization in high-dimensional space.

As shown in Fig 1, the visual feedback simulation framework works by the following steps:

- *Step 1* (Input setting): Specify the mathematical model based on the target biological pattern[2, 7, 29]. Set the adjustable model parameters and their search scopes. Define the pattern topology and quantitative pattern features of the pattern, and define the cost function to evaluate the model parameters.
- *Step 2* (Initialization): Set the target pattern topology and the quantitative pattern features based on the target biological pattern. Set the group number of the model parameters in the DE algorithm, and initialize all of the groups of model parameters randomly in the search scopes.
- *Step 3* (Numerical simulation): For each group of model parameters, numerically solve the PDE system in the mathematical model. Visualize the numerical solution data as an image that becomes the simulation result.
- *Step 4* (Feature extraction): For each simulation image, if a Turing pattern is formed, then extract the pattern topology and describe the pattern features quantitatively by the image processing methods, such as image binarization, skeleton extraction, and edge detection.
- *Step 5* (Parameter evaluation): Evaluate the model parameters by comparing the pattern topology and quantitative pattern features. The evaluation value is smaller if the topology and features are more similar to the target. The evaluation value is set to a very large value if the Turing pattern cannot be formed with these parameters.
- *Step 6* (Parameter identification): If the evaluation value is small enough or it reaches the maximum number of iterations, the simulation framework stops running and outputs the simulation result with the minimum evaluation. Otherwise, calculate groups of new model parameters by utilizing all of the groups of model parameters and their evaluations by the DE algorithm; then, go to Step 3.

It is easy to extract and compare the image features automatically by image processing methods due to the strong contrast and low noise of the simulation images; thus, the simulation framework can run completely automatically. If it is difficult to extract the image feature, such as special requirements of simulation results or 3D simulations, then Steps 4 and 5 can be replaced by experts’ scores, which makes the simulation framework semi-automatic. Either way, the simulation framework searches the model parameters nonlinearly in high-dimensional parameter space, which is a more efficient way to identify the model parameter values.

### Visual feedback simulation framework of VMCs pattern formation

In embryonic development, mesenchymal stem cells aggregate and organize into patterned tissues[27, 30]. Garfinkel et al. showed that multi-potential adult VMCs could self-organize into patterns in vitro and that these patterns could be described and predicted by a reaction-diffusion mathematical model[21]. In this paper, numerical simulations that correspond to VMC pattern formation will be developed satisfactorily by the visual feedback simulation framework.

#### Mathematical model and numerical simulation

The VMC pattern formation is modeled as the following PDE system, in which the concentrations of determined activator morphogen *U* and inhibitor morphogen *V* are distributed over a 2D domain[21].

$$\frac{\partial U}{\partial t}=D\left(\nabla^{2}U\right)+\gamma \left[\frac{U^{2}}{\left(1+kU^{2}\right)V}-cU\right]$$ (1)

$$\frac{\partial V}{\partial t}=\left(\nabla^{2}V\right)+\gamma \left[U^{2}-eV+S\right]$$ (2)

In this dimensionless mathematical model, parameter *D = DU/DV* is the ratio of the diffusion coefficients of the activator and inhibitor. Parameter *γ* is a scaling parameter that relates the chemical kinetics, the spatial domain size, and the diffusion rates. Parameter *k* in the autocatalytic term governs the saturation level of the reaction. Parameters *c* and *e* represent the first-order degradation rates of the activator and inhibitor, respectively, and *S* represents an exogenous source of inhibitor.

We perform numerical simulation of the mathematical model on a 100 × 100 grid. The PDE system is solved numerically by using the Euler method with no-flux boundary conditions. Here, we use the pattern of concentration of the activator *U* to denote the cell pattern. The solution of the variable *U* is saved as an image after image gray-scale transformation. To improve the simulation efficiency, a parallel computing technique is applied in this framework based on Graphics Processing Unit (GPU) and Compute Unified Device Architecture (CUDA) programming.

#### Implementation of the visual feedback simulation framework

There are six parameters in the mathematical model of the VMCs pattern formation. The parameters *D*, *c*, *e* have been estimated experimentally or theoretically[21]. The scaling parameter *γ*, which relates to the time scale of the biological kinetics and the length scale of the experimental domain, changes in different experiments. The value of the parameter *k* is difficult to choose. We assume that there is no inhibitor exogenous source in the experiment for simplicity. Therefore, the parameters *k* and *γ* are selected to be the adjustable parameters in the visual feedback simulation framework, whereas the other parameters are set to constants, with the values *D* = 0.005, *c* = 0.01, *e* = 0.02, and *S* = 0. The search scopes of *k* and *γ* are set to [0, 0.35] and [0, 30000], respectively, according to the Turing space analysis[29].

VMCs patterns in biological experiments include stripe, spot, and labyrinthine patterns, which are set as the pattern topologies in the simulation framework. Furthermore, the pattern-area-to-total-area ratio *R*^1^ and the perimeter-area ratio of the pattern *R*^2^ are utilized as quantitative pattern features when we consider the pattern topology and pattern scale, such as the size of the spots and the quantity of the stripes. The pattern-area-to-total-area ratio *R*^1^, which is the ratio of the area of the cell region to the area of the total region, is utilized to distinguish the pattern topologies. The perimeter-area ratio of the pattern *R*^2^, which is the ratio of the perimeter of the cell region to the area of the cell region, is utilized to describe the pattern scale, when the pattern topology has been determined. These two ratios are calculated by counting the number of pixels in the binary image or the edge image of the pattern image (See S1 Text for details of the image processing).

The cost function *f*_*VMCs*_ is defined as

$$f_{VMCs}=[{\left(R^{1}-R_{tar}^{1}\right)}^{2}+{\left(R^{2}-R_{tar}^{2}\right)}^{2}]*10^{5}$$ (3)

where $R_{tar}^{1}$ and $R_{tar}^{2}$ are the pattern-area-to-total-area ratio and perimeter-area ratio of the target pattern. The difference in the quantitative pattern features is multiplied by 10^5^ to make the evaluation value close to an integer.

### Visual feedback simulation framework of lung development

Recent experimental work in lung development has described the branching patterns, including side branching and tip bifurcation[31]. A 4-variable PDE system has been employed as a mathematical model to describe the reaction and diffusion of the morphogens in lung development, which led to the creation of lung branching patterns[32, 33]. We have also analyzed the different branching patterns and pattern switches by altering the key parameters in the model[34, 35]. In this paper, we use a visual feedback simulation framework to accomplish numerical simulations of lung branching patterns.

#### Mathematical model and numerical simulation

The branching pattern formation in the development of the lung is modeled by the following 4-variable PDE system. The variables in the first three equations are concentrations of chemical morphogens: an activator *A*, an inhibitor *H*, and a substrate chemical S; the variable *Y* in the last equation is a marker for cell differentiation (*Y* = 1 means that the cell is differentiated).

$$\frac{\partial A}{\partial t}=D_{A}\left(\nabla^{2}A\right)+\frac{cA^{2}S}{H}-\mu A+\rho_{A}Y$$ (4)

$$\frac{\partial H}{\partial t}=D_{H}(\nabla^{2}H)+cA^{2}S-\nu H+\rho_{H}Y$$ (5)

$$\frac{\partial S}{\partial t}=D_{S}(\nabla^{2}S)+c_{0}-\gamma S-\varepsilon YS$$ (6)

$$\frac{\partial Y}{\partial t}=dA-eY+\frac{Y^{2}}{1+fY^{2}}$$ (7)

In the first three equations of this model, Parameters *D*_*A*_, *D*_*H*_, *D*_*S*_ and *μ*, *v*, *γ* represent the diffusion coefficients and first-order degradation rates of the activator *A*, inhibitor *H* and substrate *S*, respectively. Parameter *c* describes the increase rate of *A* and *H* by *A* and *S*. *A* and *H* are up-regulated by differentiated cells *Y* at rates *ρ*_*A*_ and *ρ*_*H*_, respectively. *S* is produced at a constant rate *c*_0_ and is consumed by *Y* at a rate *ε*. In the last equation about *Y*, the parameters *d*, *e*, and *f* are used to adjust the cell commitment.

The formation of the branching patterns is related to the dynamics among the variables in the PDE model[36–39]. In Eqs (4)–(7), the inhibitor *H* serves to mediate the lateral inhibition, while the substrate *S* provides the directional drive for forming the straight lines. For binary branches, as the local activator (*A*) peak forms and migrates at the end of the branch tip[32], the peak expands transversely. Then the lingering inhibitor peak forces the activator peak into two daughter peaks, which leads to the branch bifurcation at the growing tip. For side branches, new activator peaks insert at the side of an existing line due to the combination of the inhibitor and the substrate. Then the side branches emerge when the attraction of the substrate overcomes the lateral inhibition. As the consumption rate of *S* by *Y* (parameter *ε*) increases, the branching pattern can switch from side branching to tip bifurcation[34].

The numerical simulation of the model is performed on a 200×200 grid, with no-flux boundary conditions, using two-step Runge-Kutta methods. The initial conditions of the simulation are *A* = 0.001, *H* = 0.01, *S* = 1, and *Y* = 0, with a uniform distribution in space. The solution of the variable *Y* in Eq (7) is directly saved as a 2D or 3D simulation image of the lung branching pattern. The simulation finishes when the growth of the lung branching pattern reaches a certain degree.

#### Implementation of the visual feedback simulation framework

There are fourteen parameters in the mathematical model of the lung branching pattern formation. Because the model has not been fully verified in a biological experiment, we select only five key parameters *D*_*H*_, *μ*, *ρ*_*H*_, *c*_0_, *ε*, which are sensitive to the branching pattern topology or features, as the adjustable parameters to test the visual feedback simulation framework. The search scopes of these parameters are set as follows: *D*_*H*_ = [0.2, 0.3], *μ* = [0.1, 0.2], *ρ*_*H*_ = [0.00005, 0.00015], *c*_0_ = [0.01, 0.1], *ε* = [0.05, 0.15]. The lung branching patterns can be formed in these parameter scopes. The other parameters are set to constants that appear as one of the parameter combinations in Ref[34]: *D*_*A*_ = 0.02, *D*_*S*_ = 0.06, *v* = 0.04, *γ* = 0.02, *c* = 0.002, *ρ*_*A*_ = 0.03, *d* = 0.008, *e* = 0.1, *f* = 10.

**Fig 2:**
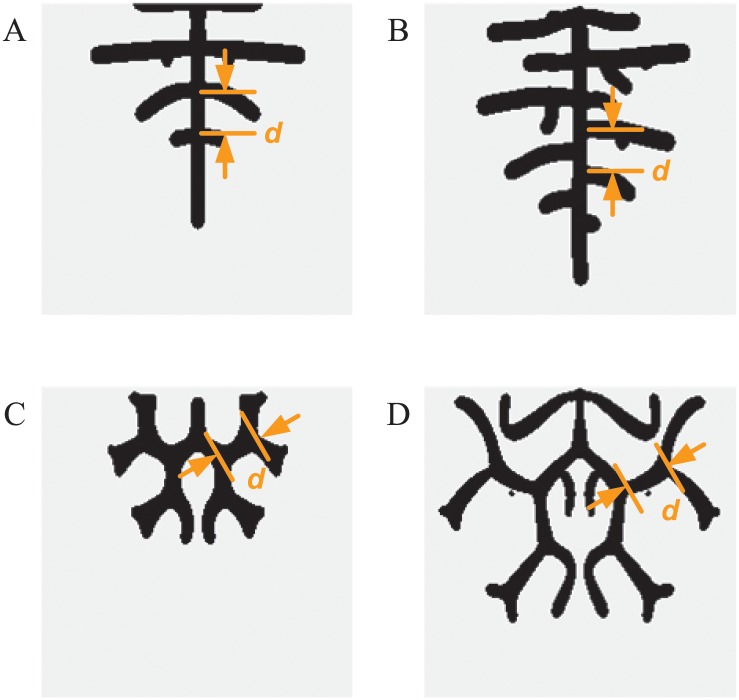
Four different lung branching patterns in simulation. (A) Zygomorphic side branching pattern. (B) Alternating side branching pattern. (C) Tip bifurcation pattern. (D) Hybrid branching pattern. There is a long main stalk in the zygomorphic side branching pattern (A) and alternating side branching pattern (B). The side branches in the zygomorphic side branching pattern are symmetric, while they grow alternatively in the alternating side branching pattern. The main stalk bifurcates into two equal-sized branches in the tip bifurcation pattern (C) and hybrid branching pattern (D). In comparison with the tip bifurcation pattern, there are side branches in the hybrid branching pattern. The quantitative pattern feature of branching pattern, the branch length *d*, is marked in pattern simulation images.

In the study of the lung development, researchers have paid more attention to the pattern topologies of the lung branching, such as domain branching or side branching and tip bifurcation[31]. Therefore, we primarily analyze the pattern topologies in the simulation framework. According to the branch characteristics in biological experiments and in previous simulations, the lung branching patterns can be divided into four approximate categories: a zygomorphic side branching pattern, alternating side branching pattern, tip bifurcation pattern and hybrid branching pattern. The simulation images are shown in Fig 2. In the zygomorphic side branching pattern and alternating side branching pattern, there is a long main stalk in the middle, and side branches grow on both sides. The zygomorphic side branching pattern is symmetric, but the alternating side branching pattern is not. In the tip bifurcation pattern and hybrid branching pattern, the main stalk bifurcates into two equal-sized branches from its apex, and similarly, each branch splits into two daughter branches. Compared with the tip bifurcation pattern, a hybrid branching pattern has side branches. These four different patterns are set as pattern topologies in the simulation framework. Furthermore, we use the branch length *d*, which is the distance between two adjacent branches, as a quantitative pattern feature (shown in Fig 2), considering the biological concerns in lung development. The pattern topologies and pattern features are extracted automatically by skeleton extraction and pixel scan. (See S1 Text for details).

The cost function *f*_*Lung*_ is defined as

$$f_{Lung}=\delta +{\left(d-d^{Tar}\right)}^{2}$$ (8)

$$\delta =\{\begin{array}{cc} 0 & if\;PT=PT^{Tar}\\ 500000 & if\;PT\neq PT^{Tar} \end{array}$$ (9)

where *d*^*Tar*^ represents the branch length of the target pattern and *PT* and *PT*^*Tar*^ represent the pattern topologies of the simulation result and the target pattern. Here, *δ* represents similarity of the pattern topologies. If the pattern topologies of the simulation result and the target pattern are different, then the cost function is set to a very large value; if they are the same, the cost function is calculated according to the quantitative pattern feature.

## Results

### Simulation results of VMCs based on the visual feedback simulation framework

**Fig 3:**
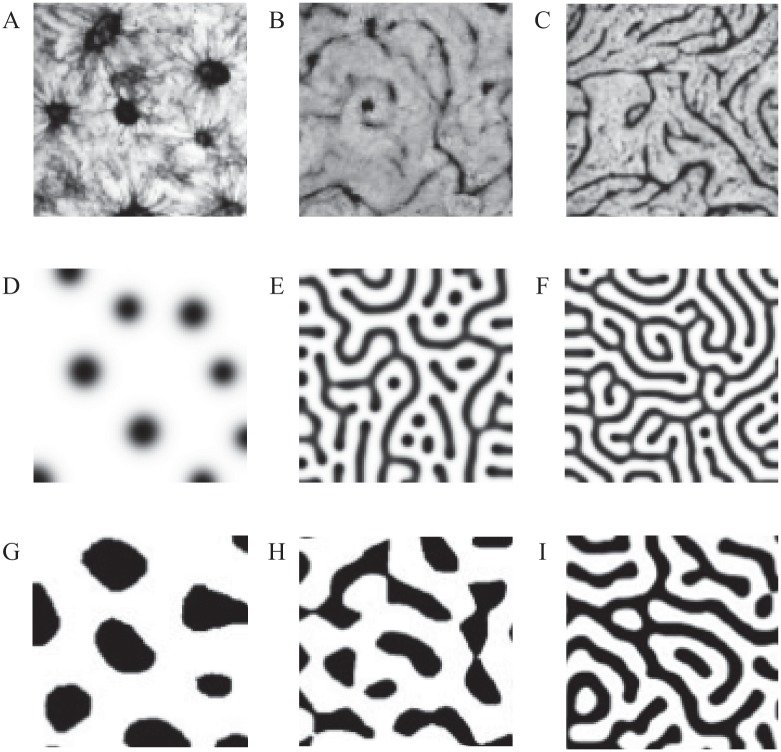
Different VMC patterns in biological experiments[21] and in simulations. (A, D, G) Spot pattern. (B, E, H) Stripe pattern. (C, F, I) Labyrinthine (stripe doubling) pattern. The first line (A-C) shows the target patterns of the VMCs. The second line (D-F) shows the corresponding simulation patterns from the visual feedback simulation framework in this paper. The third line (G-I) shows the corresponding simulation results in Ref[21]. (A-C) and (G-I) are cropped for display, but the image scales are retained. The pattern topologies of both types of simulation are the same as those of biological patterns. However, the quantitative pattern features of the simulation results from the simulation framework are much more similar to the biological patterns than the results of the manual simulation.

We set the VMC patterns in the first line of Fig 3 (Fig 3A–3C) to be the target patterns, and we use the visual feedback simulation framework to perform the simulation. The quantitative pattern features *R*^1^ and *R*^2^ of the target patterns are calculated first, as shown in Table 1. Then, each *R*^1^,*R*^2^ combination is set as a simulation target, and the simulation framework obtains the unknown model parameters and the simulation pattern. The second line of Fig 3 (Fig 3D–3F) shows the simulation images that correspond to three types of VMC patterns in biological experiments. The values of the unknown model parameters, the quantitative pattern features in the simulation images and the evaluation values of the cost function as well as the number of simulations are listed in Table 2.

**Table 1:** Quantitative pattern features of the VMC patterns.

Pattern	*R*^1^	*R*^2^
Spot pattern	0.22	0.31
Stripe pattern	0.40	0.56
Labyrinthine pattern	0.46	0.67

**Table 2:** Simulation results of the VMC patterns.

Pattern	Values of model parameters		Quantitative pattern features		Evaluation value	Simulation number
	*k*	*γ*	*R*^1^	*R*^2^		
Spot pattern	0.01	1136	0.2017	0.3044	37	152
Stripe pattern	0.15	14401	0.4145	0.5469	38	35
Labyrinthine pattern	0.2	21375	0.4524	0.6397	98	53

In Fig 3, the simulation images (Fig 3D–3F) from the simulation framework are compared with the target biological patterns (Fig 3A–3C) and the simulation results in Ref[21] (Fig 3G–3I). The pattern topologies of both types of simulations are the same as those of biological patterns. However, for some of the pattern features, e.g., the sizes of the spots in the spot pattern or the widths of the stripes in the stripe pattern and labyrinthine pattern, the simulation results from the simulation framework are much more similar to biological patterns than the results from the manual simulation because the simulation framework uses both the pattern topology and the pattern features to perform the simulation. Compared to Tables 1 and 2, the quantitative pattern features *R*^1^ and *R*^2^ of the simulation images are close to those of the target patterns. The simulation results demonstrate that the simulation framework can achieve the pattern formation simulation by VMCs and obtain more similar simulation results than the manual method.

**Fig 4:**
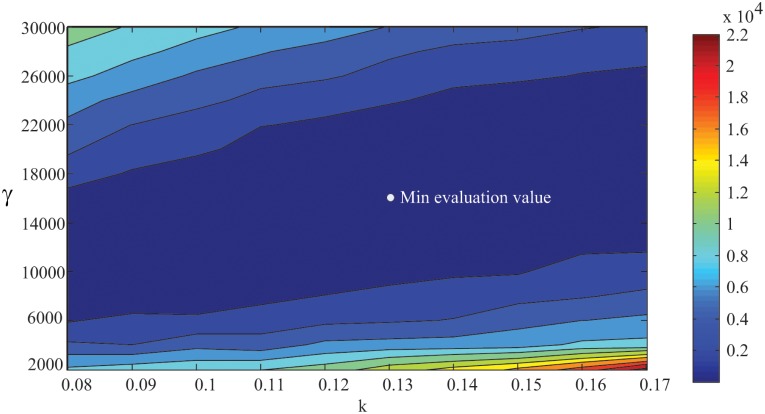
The evaluation values of the model parameters *k* and *γ* in their search scopes. The minimum evaluation value 22 (marked as white point) is obtained when *k* = 0.13 and *γ* = 16000, which are similar to the results of the visual feedback simulation framework with *k* = 0.15 and *γ* = 14401.

The simulation patterns of all types of biological patterns are obtained in a finite number of iterations in the simulation framework. It takes more time for the spot pattern simulation because the values of the unknown model parameters are close to the lower limits of the search scopes. If we perform simulation in a tradition way, we must traverse the search scope of every parameter. Taking the stripe pattern as an example, if the search scopes of the model parameters *k* and *γ* are set to [0.08, 0.17] and [2000, 30000] and the sampling intervals are 0.01 and 2000, respectively, we need a total of 10*15 = 150 simulations. Fig 4 shows the evaluation values of the parameter combinations. We obtain the minimum evaluation value (= 22) when *k* and *γ* are 0.13 and 16000, which are similar to the results of the visual feedback simulation framework. However, we perform only 35 simulations to obtain the result. It can be seen that the simulation framework provides a more efficient way to perform simulations because the model parameters are searched nonlinearly in a high-dimensional parameter space.

### Simulation results of lung branching based on the visual feedback simulation framework

**Fig 5:**
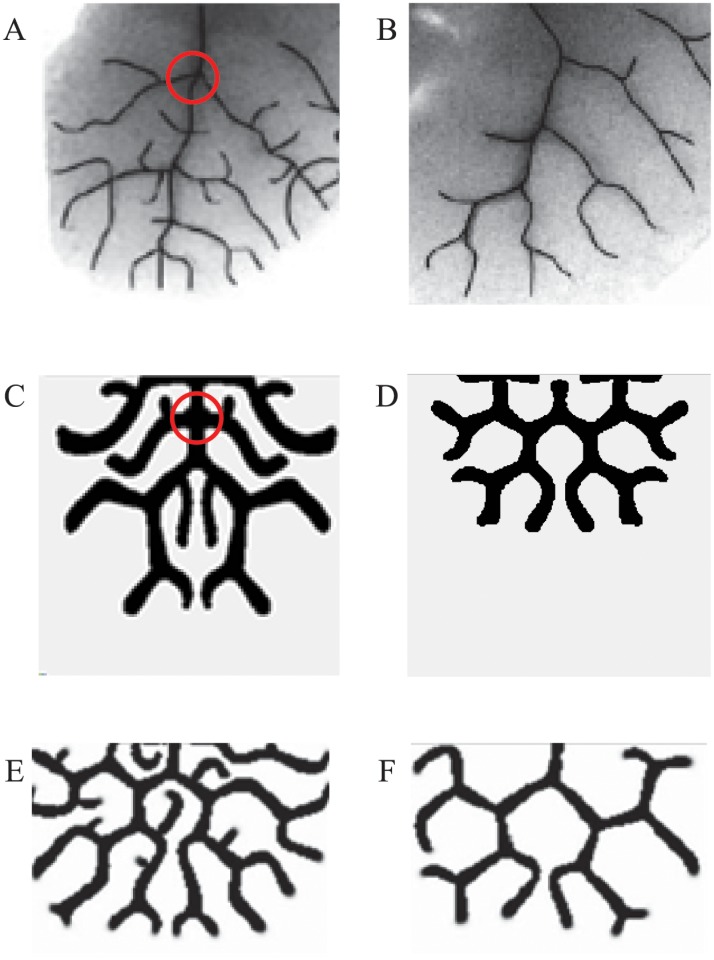
Lung branching patterns in biological experiments[33] and in simulations. (A, C, E) Normal branching pattern. (B, D, F) Branching pattern without side branching. The first line (A and B) shows the target patterns of the lung branching. The second line (C and D) shows the corresponding simulation patterns of the simulation framework. The third line (E and F) shows the corresponding simulation results in Ref[33]. For the branching patterns without side branching, both of the simulation results (D and F) are similar to the biological branching pattern (B). For the normal branching pattern, both of the simulation results (C and E) have side branching, but the branching topology of the simulation results using the simulation framework (C) is more similar to that of the biological pattern (A), as shown by the marked branching structures.

For the target patterns, we use the pulmonary vascular patterns in lungs in Ref[33], including normal branching pattern in the lung of wild type mouse and branching pattern that lacks side branching in MGP transgenic lung (Fig 5A and 5B). The normal branching pattern and branching pattern without side branching are classified as the hybrid branching pattern and tip bifurcation pattern respectively, and the quantitative pattern features *d* are set to 15 and 20 pixels. We use the visual feedback simulation framework to perform the simulations. Fig 5C and 5D and Table 3 show the corresponding simulation patterns and simulation results.

**Table 3:** Simulation results of the lung branching pattern.

Pattern	Values of model parameters					*d*	Evaluation value	Simulation number
	*D*_*H*_	*μ*	*ρ*_*H*_	*c*_0_	*ε*			
normal branching pattern	0.2135	0.1514	6.8×10^−5^	0.023	0.321	14	1	270
branching pattern lack of side branching	0.384	0.153	4.8×10^−4^	0.151	1.018	18	4	180

In Fig 5, the simulation images (Fig 5C and 5D) from the simulation framework are compared with the branching patterns in the biological experiments (Fig 5A and 5B) and the simulation results in Ref[33] (Fig 5E and 5F). For the branching pattern without side branching, both of the simulation results (Fig 5D and 5F) are similar to the biological branching pattern (Fig 5B), and it is relatively easy to find the results. As shown in Table 2, the simulation framework obtains the proper branching pattern after approximately 180 simulations. For the normal branching pattern, both of the simulation results (Fig 5C and 5E) have side branching. However, the branching topology of the simulation result using the simulation framework is more similar to that of the biological pattern, such as the marked branching structures in Fig 5A and 5C. Furthermore, there are many parameters in the mathematical model of lung branching, and we found that the normal branching pattern with side branching is very sensitive to the model parameters in the simulation. It is difficult and time-consuming to find this pattern by traversing the search scope of every parameter with a fixed sampling interval. However, by using the simulation framework, the simulation loop of the normal branching pattern ends in approximately 270 iterations, which saves a large amount of time.

**Fig 6:**
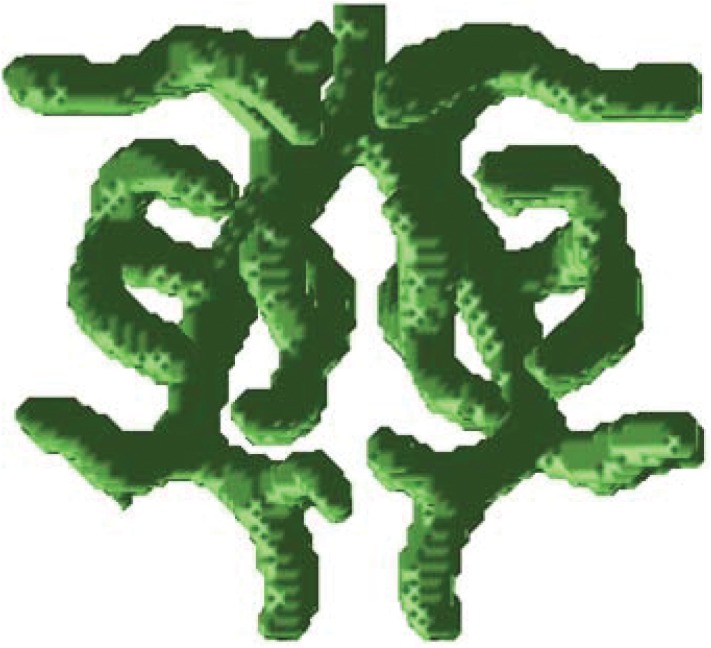
3D simulation result by using the simulation framework. The branching pattern that has only horizontal branches and less branching is set as the simulation target. The corresponding model parameters are calculated semi-automatically based the experts’ scores.

We attempt to perform 3D simulations of lung branching using the simulation framework. Two key parameters, *ρ*_*H*_ and *c*_0_, are chosen as unknown model parameters for simplicity. We evaluate each parameter combination manually by giving a score to each 3D simulation result due to the difficulties of 3D image processing. The branching pattern that has only horizontal branches and less branching is set as the simulation target. As shown in Fig 6, the simulation framework obtains the proper simulation result after approximately 90 simulations, with the optical parameter values *ρ*_*H*_ = 0.00072, = 0.126. The 2D and 3D simulation results indicate that this simulation framework can achieve the pattern formation simulation for lung branching.

## Discussion

Pattern formation in developing biological systems is controlled by genes. Mathematical modelling can be used to describe and predict the essential steps in the processes. Because the interactions of these complex processes are usually nonlinear, the mathematical models of pattern formation are usually PDE systems. A numerical solution of a PDE system depends on the parameters of the equations. It is important and time-consuming to choose the appropriate values of the parameters to obtain the appropriate simulation result. In this paper, a visual feedback simulation framework is proposed to solve the problem of model parameter identification in biological pattern formation simulation. With the basic principle of feedback control, the simulation framework visualizes the simulation result and extracts the image features, including the pattern topology and pattern features, as system feedback. Then, the unknown parameters of the mathematical model are calculated, according to the differences between the image features of the simulation image and the target biological pattern.

Considering two typical applications, the visual feedback simulation framework is utilized to accomplish satisfactory pattern formation simulations for VMCs and lung development. Many different patterns, including the spot, stripe, and labyrinthine patterns of VMCs, the normal branching pattern and branching pattern without side branching for lung branching are obtained by the simulation framework in a finite number of iterations, which verifies the effectiveness and flexibility of the simulation framework.

The simulation framework extracts the pattern topology and pattern features by image processing and compares those features automatically, which enables it to overcome some of the weaknesses of manual image comparison, such as quantitative comparisons (e.g., the size of the spots in a spot pattern or the width of the stripes in a stripe pattern and the labyrinthine pattern of the VMCs) and detailed comparisons (e.g., the branching structure in the normal branching pattern in lung). We obtain more objective simulation results in the visual feedback simulation framework by defining an appropriate cost function to evaluate the image differences between the simulated image and the target pattern. At the same time, the simulation framework can have the advantage of manual simulation by combining the experts’ scores, to enable us to obtain better simulation results.

Whether using a cost function or experts’ scores, the simulation framework searches the model parameters nonlinearly in a high-dimensional parameter space. This approach is a more efficient way to achieve simulation targets, especially simulation patterns that are sensitive to the model parameters, compared to using traditional manual simulation, in which the parameters are traversed linearly in parameter space. Furthermore, the simulation framework is easy to expand to other types of biological pattern formation by using different mathematical models and extracting different pattern features. Numerical simulation of biological pattern formation is greatly improved by using the visual feedback simulation framework.

In the process of simulation, we obtain various types of simulation patterns in given parameter spaces. These simulation patterns can help us understand the mathematical model and discover new phenomena in biological pattern formation. Furthermore, delay feedback is widely existed in the real world. For example, time delay and spatial diffusion in the mathematical model are utilized to explain the herbivore outbreak[40–42]. We can analysis the phenomena in deep by using the simulation framework.

It is found that the convergence speed of the simulation is seriously affected by the search scopes and the initial values of the parameters in the DE algorithm. In the future, we will focus on the search algorithm for global optimization and feature extraction for various types of biological pattern formation and expand the application of the visual feedback simulation framework, which will accelerate the verification and selection of the mathematical model and promote research on biological pattern formation mechanisms.

## Supporting information

S1 Code Numerical simulation of VMCs by a visual feedback simulation framework. The simulation framework of the VMCs is implemented in CUDA for GPU implementation.
(PDF)

S2 Code Numerical simulation of lung branching by a visual feedback simulation framework. The simulation framework of lung branching is implemented in CUDA for GPU implementation.
(PDF)

S1 Text Pattern topology and pattern feature extractions. The quantitative pattern features of VMCs, the pattern topologies and quantitative pattern features of lung branching are extracted by image processing automatically. We show the image processing method in the S1 Text.
(DOCX)
