# Supplementary material for: Automated numerical simulation of biological pattern formation based on visual feedback simulation framework
Source: PLoS One. 2017 Feb 22;12(2):e0172643. doi: 10.1371/journal.pone.0172643 (PMC5321435; doi:10.1371/journal.pone.0172643)
Supplement: S1 Code — The simulation framework of the VMCs is implemented in CUDA for GPU implementation. (PDF) [file pone.0172643.s001.pdf]

```
#include <cstdio>
#include <cmath>
#include <cstdlib>
#include <cstring>
#include <sys/stat.h>
#include "CImg.h"
#include <unistd.h>
#include <sys/types.h>
#include <fcntl.h>
#include "time.h"

using namespace std;
using namespace cimg_library;

//================Simulation Target==============================================
#define AREA_TAR 0.40
#define CIRL_AREA_TAR 0.56 // Stripe pattern

//===============Simulation=========================================

#define XLEN 	100
#define YLEN 	XLEN

#define DELTA_TAU 5E-6
#define TAU 			1
#define PIC 			20

#define D 	0.005
#define C 	0.01
#define E 	0.02
#define S 	0

#define UINIT 	1.8694
#define VINIT 	174.7238

// Grid array
float ha0[XLEN][YLEN];
float ha1[XLEN][YLEN];
float ha2[XLEN][YLEN];
float ha3[XLEN][YLEN];
char	dirc[256];
FILE * f;
```

```
typedef float ( * mat_t )[YLEN];
__global__ void update ( mat_t dud, mat_t duu, mat_t dvd, mat_t dvu, float K, int GAMMA ) {
        int i, j;
        i = blockIdx.x * blockDim.x + threadIdx.x;
        j = blockIdx.y * blockDim.y + threadIdx.y;

        if ( i <= 0 || i >= XLEN - 1 || j <= 0 || j >= YLEN - 1 ) return;

        duu[i][j] = dud[i][j] +( D * (      ( dud[i - 1][j] - 2 * dud[i][j] + dud[i + 1][j] )* XLEN * XLEN +
                                            ( dud[i][j - 1] - 2 * dud[i][j] + dud[i][j + 1] )* YLEN * YLEN ) +
                                         GAMMA * ( ( dud[i][j] * dud[i][j] ) / ( 1 + K * dud[i][j] * dud[i][j] ) /
dvd[i][j] - C * dud[i][j] )
                        ) * DELTA_TAU;
        dvu[i][j] = dvd[i][j] + (      (      ( dvd[i - 1][j] - 2 * dvd[i][j] + dvd[i + 1][j] )* XLEN * XLEN +
                                                ( dvd[i][j - 1] - 2 * dvd[i][j] + dvd[i][j + 1] )* YLEN * YLEN     )
+
                                        GAMMA * ( dud[i][j] * dud[i][j] - E * dvd[i][j] + S )
                        ) * DELTA_TAU;
        __syncthreads ( );

        if ( i == 1 && j == 1 ) {
                duu[0][0] = duu[1][0] = duu[0][1] = duu[1][1];
                dvu[0][0] = dvu[1][0] = dvu[0][1] = dvu[1][1];
        } else if ( i == 1 && j == YLEN - 2 ) {
                duu[0][YLEN-1] = duu[1][YLEN-1] = duu[0][YLEN-2] = duu[1][YLEN-2];
                dvu[0][YLEN-1] = dvu[1][YLEN-1] = dvu[0][YLEN-2] = dvu[1][YLEN-2];
        } else if ( i == XLEN - 2 && j == 1 ) {
                duu[XLEN-1][0] = duu[XLEN-2][0] = duu[XLEN-1][1] = duu[XLEN-2][1];
                dvu[XLEN-1][0] = dvu[XLEN-2][0] = dvu[XLEN-1][1] = dvu[XLEN-2][1];
        } else if ( i == XLEN - 2 && j == YLEN - 2 ) {
                duu[XLEN-1][YLEN-1]   =   duu[XLEN-2][YLEN-1]   =   duu[XLEN-1][YLEN-2]   =   duu[XLEN-
2][YLEN-2];
                dvu[XLEN-1][YLEN-1]   =   dvu[XLEN-2][YLEN-1]   =   dvu[XLEN-1][YLEN-2]   =   dvu[XLEN-
2][YLEN-2];
        } else if ( i == 1 || j == 1 || i == XLEN - 2 || j == YLEN - 2 ) {
                if ( i == 1 ) {
                        duu[0][j] = duu[1][j];
                        dvu[0][j] = dvu[1][j];
                } else if ( j == 1 ) {
                        duu[i][0] = duu[i][1];
                        dvu[i][0] = dvu[i][1];
                } else if ( i == XLEN - 2 ) {
                        duu[XLEN-1][j] = duu[XLEN-2][j];
```

```
                dvu[XLEN-1][j] = dvu[XLEN-2][j];
            } else if ( j == YLEN - 2 ) {
                duu[i][YLEN-1] = duu[i][YLEN-2];
                dvu[i][YLEN-1] = dvu[i][YLEN-2];
            }
        }

}
//==========================End Simulation===============================


float cout_result ( int n, float ( * dp )[YLEN],const char* type ) {
        int i, j;
        float min, max;
        char file [256];
        float evaluate=0.0f;

        float * d = &dp[0][0];
        float * de = d + XLEN * YLEN;
        min = max = dp[0][0];

        float area=0;               //===================================Area
        float cirl=0;                //===================================Perimeter
        CImg < char > dst( XLEN, YLEN );
        CImg < char > dst_1( XLEN, YLEN );

        for ( ++d; d != de; ++d ) {
                if ( min > *d ) min = *d;
                if ( max < *d ) max = *d;
        }

        fprintf (f, "*** min = %.5f    max = %.5f *****\n", min, max );

        if (n == PIC)             // show last image
        {
                float tran_factor = (max - min)/255;
                CImg < char > img ( XLEN, YLEN);

                for( i = 0; i < XLEN; ++i ) {
                        for ( j = 0; j < YLEN; ++j ) {
                                int b = 255 - ( dp[i][j] - min) / tran_factor;

                                if(    b <180 )
                                {
```

```
                    dst(i,j) = 0;
                    area++;
                }
                else
                {
                    dst(i,j) = 255;
                }
                dst_1(i,j)=dst(i,j) ;
                img ( i , j ) = b;
            }
        }
        sprintf( file ,"%s.bmp", dirc );
        img.save_bmp ( file );

        if(max-min<0.02)            // no pattern
        {
            evaluate = 50000;
            printf ( " evaluate = %.3f\n", evaluate );
            return evaluate ;
        }



        for(i=1;i<YLEN-1;i++)
        {
            for(j=1;j<XLEN-1;j++)
            {
                if(dst(j,i)==0&&dst(j-1,i)==0&&dst(j+1,i)==0&&dst(j-1,i-1)==0&&dst(j,i-
1)==0&&dst(j+1,i-1)==0&&dst(j-1,i+1)==0&&dst(j,i+1)==0&&dst(j+1,i+1)==0)
                    dst_1(j,i)=255;
            }
        }
        for(i=0;i<YLEN;i++)
        {
            for(j=0;j<XLEN;j++)
            {
                if(dst_1(j,i)==0)
                {
                    cirl++;
                    dst_1(j,i)=255;
                }
                else
                    dst_1(j,i)=0;
            }
```

```
		}


		float area_ratio=(float)area/XLEN/YLEN;		//Pattern area-total area ratio
		float cirl_area_ratio=(float)cirl/area;			//Perimeter-area ratio of the pattern

		evaluate=100000*((AREA_TAR-area_ratio)*(AREA_TAR-area_ratio)+(CIRL_AREA_TAR-
cirl_area_ratio)*(CIRL_AREA_TAR-cirl_area_ratio));

		printf ( " evaluate = %.3f\n", evaluate );
		printf ( " area_Ratio= %.3f\n", area_ratio );
		printf ( " ed_ar_ratio= %.3f\n", cirl_area_ratio );


		sprintf( file ,"%s	evalu=%.4f.bmp", dirc,evaluate );
		dst_1.save_bmp ( file );

		return evaluate ;
	}
	return -1;
}



//=====================Cost Function=====================================
float evaluate(float* vector)
{
	float evaluate=0.0f;

	int i, j, t;
	clock_t tb, te;

	float K = vector[0];
	int GAMMA = (int)vector[1];

	long STEP = TAU / DELTA_TAU;
	int step = STEP / PIC;

	float ( * hu )[YLEN] = ha0;
	float ( * hv )[YLEN] = ha1;

	int memsize = XLEN * YLEN * sizeof ( float );
	float ( *dud )[YLEN];
	cudaMalloc ( ( void ** )&dud, memsize );
```

```
	float ( *duu )[YLEN];
	cudaMalloc ( ( void ** )&duu, memsize );
	float ( *dvd )[YLEN];
	cudaMalloc ( ( void ** )&dvd, memsize );
	float ( *dvu )[YLEN];
	cudaMalloc ( ( void ** )&dvu, memsize );

	float ( *gtmp)[YLEN];

	srand48 ( time ( NULL ) );

	//sprintf ( dirc, "K=%.2f    GAMMA=%d",K,GAMMA );
	//printf( "\n\n************** para = %s *****************\n",dirc );
	// mkdir ( dirc, 0700 );

		tb = clock ( );

	//printf(" \n\nHomogenous activator and inhibitor semistable concentration :\n " );
	//printf(" activator : %2.4f      inhibitor : %2.4f \n\n",UINIT,VINIT );

	for(i = 0;i < XLEN;i++ ) {
		for( j = 0; j < YLEN; j++ ){
			hu[i][j] = UINIT* ( 1.0 + 0.04 * drand48() - 0.02 );
					hv[i][j] = VINIT* ( 1.0 + 0.04 * drand48() - 0.02 );
			}
	}

	cudaMemcpy ( dud, hu, memsize, cudaMemcpyHostToDevice );
	cudaMemcpy ( dvd, hv, memsize, cudaMemcpyHostToDevice );

	// update
#define BLOCK_SIZE	32
	dim3 dimBlock ( BLOCK_SIZE, BLOCK_SIZE );
	dim3 dimGrid	( ( XLEN + BLOCK_SIZE - 1 ) / BLOCK_SIZE, ( YLEN + BLOCK_SIZE - 1 ) /
BLOCK_SIZE );


	// file to record max & min of U
	char data[256];
	sprintf( data ,"%s.txt", dirc );
	f = fopen( data,"w" );

	for ( t = 0; t <= STEP ; ++t ) {
		update <<< dimGrid, dimBlock >>> ( dud, duu, dvd, dvu,K,GAMMA );
```

```
		cudaMemcpy ( hu, dud, memsize, cudaMemcpyDeviceToHost );
		cudaMemcpy ( hv, dvd, memsize, cudaMemcpyDeviceToHost );
		gtmp = dud; dud = duu; duu = gtmp;
		gtmp = dvd; dvd = dvu; dvu = gtmp;

		int pic = t % step;
		if ( pic ) continue ;
		//printf( "\n\n************** pic = %d *****************\n",t/step );
		evaluate = cout_result( t / step, hu ,"u");
		// cout_result( t / step, hv ,"v");
	}

	fclose(f);

	te = clock ( );
	printf ("GPU version time %.6lf\n", double ( te - tb ) / CLOCKS_PER_SEC );


	cudaFree ( dud );
	cudaFree ( duu );
	cudaFree ( dvd );
	cudaFree ( dvu );

	return evaluate;

}


//=================DE algorithm============================================
#define	gen_max		50
#define	NP			16
#define	DE_D		2
#define	CR			0.3f
#define	DE_F		0.6f

//=================================================================
int main( int argc, char const *argv[] )
{

	float target[NP][DE_D],mutant[NP][DE_D],trial[DE_D];
	int count=-1;
	int r1,r2,r3,q,w;
	float cost[NP],score=1;
	bool	DE_idx=0;
```

```
        float mid[DE_D];

        for(q=0;q<NP;q++)
        {
                srand48 ( time ( NULL ) );

                target[q][0]=drand48()*0.35f+0.0005f;                                          //K
                target[q][1]=(int)(drand48()*30000+1000);                     //GAMMA

                for(w=0;w<DE_D;w++)
                        mid[w]=target[q][w];



                sprintf ( dirc, "gen=%d    K=%.2f    GAMMA=%f",count,mid[0],mid[1] );
                printf( "\n\n************** para : %s *****************\n",dirc );

                        cost[q]=evaluate(mid);           //Evaluate the first generation

                if(cost[q]==0)
                {
                      for(w=0;w<DE_D;w++)
                              trial[w]=target[q][w];
                      DE_idx=1;
                      break;
                }
        }
        count++;

        int i,j;
        while(count<gen_max&&DE_idx==0)
        {
                for(i=0;i<NP;i++)
                {
                        srand((unsigned)time(NULL));
                        do r1=rand()%NP; while(r1==i);                                    //Randomly    pick    3
indexes,
                        do r2=rand()%NP; while(r2==i||r1==r2);                            //integer,mutually
different
                        do r3=rand()%NP; while(r3==i||r2==r3||r1==r3); //and all different from i
                        j=rand()%DE_D;
                        for(int k=1;k<=DE_D;k++)
                        {
                              if((rand()%100)*0.01f<CR||k==DE_D) trial[j]=target[r3][j]+DE_F*(target[r1][j]-
target[r2][j]);
```

```
                else    trial[j]=target[i][j];
                j=(j+1)%DE_D;
            }
                        if(trial[0]<=0) trial[0] = 0.01;
            if(trial[1]<=0) trial[1] = 2000;
            sprintf ( dirc, "gen=%d    K=%.2f    GAMMA=%f",count,trial[0],trial[1] );
            printf( "\n\n************** para : %s *****************\n",dirc );
            score=evaluate(trial);


        /*-----------------------------if find the optimal solution----------------------------*/
            if(score<100)
            {
                DE_idx=1;
                break;
            }
          /*--------------------------------------------------------------------------*/
            if(score<=cost[i])
            {
                for(j=0;j<DE_D;j++)    mutant[i][j]=trial[j];
                cost[i]=score;
            }
            else      for(j=0;j<DE_D;j++)    mutant[i][j]=target[i][j];
        }    //end for

                if(DE_idx) {printf("\n----Oops----\n");break;}

        for(i=0;i<NP;i++)        //After each generation,
        {                                        //move mutant vector into target vector.
            for(j=0;j<DE_D;j++)
                target[i][j]=mutant[i][j];
        }
            count++;
    } //End while.

    printf("\n---------------------------------------\n");
    for(i=0;i<NP/5;i++)
printf("\n%f    %f    %f    %f    %f\n",cost[i],cost[i+1],cost[i+2],cost[i+3],cost[i+4]);
    printf("\ncount=%d\n",count);

    if(!DE_idx)
    {
        float cost_min=cost[0];
        int minNum=0;
```

```
            for(i=1;i<NP;i++)
            {
                if(cost[i]<cost_min)
                {
                    cost_min=cost[i];
                    minNum=i;
                }
            }
            for(j=0;j<DE_D;j++)
                trial[j]=target[minNum][j];
        }
        printf("\n--------------Output----------------------\n");
        printf("K=%f**GAMMA=%f\n",trial[0],trial[1]);

    return 0;
}
```
