# Supplementary material for: Automated numerical simulation of biological pattern formation based on visual feedback simulation framework
Source: PLoS One. 2017 Feb 22;12(2):e0172643. doi: 10.1371/journal.pone.0172643 (PMC5321435; doi:10.1371/journal.pone.0172643)
Supplement: S2 Code — The simulation framework of lung branching is implemented in CUDA for GPU implementation. (PDF) [file pone.0172643.s002.pdf]

```
#include <cstdio>
#include <cmath>
#include <cstdlib>
#include <cstring>
#include <sys/stat.h>
#include "CImg.h"
#include <unistd.h>
#include <sys/types.h>
#include <fcntl.h>
#include "time.h"

using namespace std;
using namespace cimg_library;

//===============Image processing information===================================

int C_feature_mode=2;          //pattern topology：  1，alternating；2，zygomorphic；3，tip bifurcation；4，hybrid
int C_feature_d=26;                  // branch length for alternating and zygomorphic
int C_feature_length=16;             // branch length for tip bifurcation and hybrid
int C_feature_cebranch=1;            // side branching
int C_feature_branch=1;              // side branching


int feature_mode=0;
int feature_width=0;
int feature_d=0;
int feature_length=0;
int feature_cebranch=0;
int feature_branch=0;

//=================================================================

void FindBranch(CImg < unsigned char > src,int& row,int& col)
{
        int k=row; int l=col;
        int num=0;
        int index=1;
        if(src(l-1,k+1)==255)
        {
               num++;
               k=k+1; l=l-1;
        }
        else if(src(l-1,k)==255)
```

```
        {
                num++;
                k=k; l=l-1;
        }
        else if(src(l,k+1)==255)
        {
                num++;
                k=k+1; l=l;
        }
        while(index&&num<10)
        {
                index=0;
                if(src(l-1,k-1)==255) {break;}
                if(src(l-1,k+1)==255)
                {
                        if(src(l,k+1)+src(l,k+2)==255*2&&num>5) break;
                        if(src(l-1,k)==255&&src(l-2,k-1)==255&&num>5)    break;
                        num++;
                        index=1;
                        k=k+1; l=l-1;
                        continue;
                }
                else if(src(l-1,k)==255)
                {
                        if(src(l,k+1)==255&&num>5) break;
                        num++;
                        index=1;
                        k=k; l=l-1;
                        continue;
                }
                else if(src(l,k+1)==255)
                {
                        if(src(l,k+2)+src(l,k+4)==255*2&&num>5) break;
                        num++;
                        index=1;
                        k=k+1; l=l;
                        continue;
                }
        }//end while
        row=k;col=l;
}//end FindBranch

//==========================================================================
```

```
void Down_keepgoing(CImg < unsigned char > src,int row,int col)
{
	int k=row; int l=col;
	int count=0;
	int M_idx=1;
	int index=255;
	while(M_idx||index<255*3&&count<50)
	{
		count++;
		if(src(l,k+1)==255)
		{
			if(src(l-1,k)+src(l-3,k)==255*2)
			{feature_mode=4; feature_branch=1; break;}
			if(src(l+1,k)+src(l+3,k)==255*2)
			{feature_mode=4; feature_branch=1; break;}
			k=k+1; l=l;
		}
		else if(src(l-1,k+1)==255)
		{
			if(src(l-1,k)+src(l-3,k)==255*2)
			{feature_mode=4; feature_branch=1; break;}
			if(src(l+1,k)+src(l+3,k)==255*2)
			{feature_mode=4; feature_branch=1; break;}
			k=k+1; l=l-1;
		}
		else if(src(l+1,k+1)==255)
		{
			if(src(l-1,k)+src(l-3,k)==255*2)
			{feature_mode=4; feature_branch=1; break;}
			if(src(l+1,k)+src(l+3,k)==255*2)
			{feature_mode=4; feature_branch=1; break;}
			k=k+1; l=l+1;
		}
		M_idx=src(l,k+5)+src(l-1,k+5)+src(l+1,k+5);
		index=src(l,k)+src(l+1,k)+src(l-1,k)+src(l-1,k+1)+src(l+1,k+1);
	}//end while
}//end

//==============================================================================

void Up_keepgoing(CImg < unsigned char > src,int row,int col)
{
	int k=row; int l=col;
	int count=0;
```

```
		int index1=255;
		int index2=255;
		if(src(l,k+1)==255)
		{
			k=k+1; l=l;
		}
		while(index1<255*2&&index2<255*2&&count<50)
		{
			count++;
			if(src(l-1,k-1)==255)
			{
				k=k-1; l=l-1;
			}
			else if(src(l-1,k)==255)
			{
				k=k; l=l-1;
			}
			else if(src(l,k-1)==255)
			{
				k=k-1; l=l;
			}
			if(src(l,k+1)+src(l,k+2)+src(l-1,k+1)==255*3)
			{feature_mode=4; feature_branch=1; break;}

			index1=src(l,k-1)+src(l-1,k+1)+src(l-1,k-1);
			index2=src(l,k-1)+src(l,k+1)+src(l-1,k-1);
		}
}

//========================Simulation=====================================

#define RHO_A		0.03
#define D			0.008

#define XLEN		200//
#define YLEN		200//

#define D_A			0.02
#define D_S			0.06

#define	C			0.002
#define NU			0.04	//v
#define GAMMA		0.02
#define E		0.1
```

```
#define F               10


#define DELTA_X          0.3
#define DELTA_TAU DELTA_X*DELTA_X*0.4*D_H

#define TSTEP       2000
#define TAU                     (TSTEP*350)

float fa[XLEN][YLEN];
float fh[XLEN][YLEN];
float fs[XLEN][YLEN];
float fy[XLEN][YLEN];

float C_O;
float RHO_H;
float EPSILON;
float MU;
float D_H;

typedef float ( * mat_t )[YLEN];

__device__ float laplace_2( mat_t A, int i, int j , int L, int R, int Z){
    float t = 0.0f, dx = DELTA_X, dy = (DELTA_X*0.8);
    if ( i > L ) t+= ( A[i-1][j] - A[i][j] )/dx/dx;
    if ( i < R ) t+= ( A[i+1][j] - A[i][j] )/dx/dx;
    if ( j > 0 ) t+= ( A[i][j-1] - A[i][j] )/dy/dy;
    if ( j < Z ) t+= ( A[i][j+1] - A[i][j] )/dy/dy;
    return t;
}
__device__ void equation( float a, float h, float s, float y, float &da,float &dh, float& ds, float& dy,
    float C_O, float RHO_H, float EPSILON, float MU)
{
    da = C * a * a * s /h - MU * a + RHO_A * y;
    dh = C * a * a * s - NU * h + RHO_H * y;
    ds = C_O - GAMMA * s - EPSILON * y * s;
    dy = D * a - E * y + y * y / ( 1 + F * y * y );
}
__global__ void update ( mat_t dad, mat_t dau, mat_t dhd, mat_t dhu, mat_t dsd, mat_t dsu,
mat_t dyd, mat_t dyu,int L,int R,int Z, int t,
    float C_O, float RHO_H, float EPSILON, float MU, float D_H) {
    int i, j;
    i = blockIdx.x * blockDim.x + threadIdx.x;
    j = blockIdx.y * blockDim.y + threadIdx.y;
```

```
	if ( i < L || i > R || j < 0 || j > Z ) return;
	float da,dh,ds,dy;
	equation( dad[i][j], dhd[i][j], dsd[i][j], dyd[i][j], da, dh, ds, dy, C_O, RHO_H, EPSILON, MU);
	dau[i][j] = dad[i][j] + DELTA_TAU * ( da + D_A * laplace_2( dad, i, j ,L,R,Z) );
	dhu[i][j] = dhd[i][j] + DELTA_TAU * ( dh + D_H * laplace_2( dhd, i, j ,L,R,Z) );
	dsu[i][j] = dsd[i][j] + DELTA_TAU * ( ds + D_S * laplace_2( dsd, i, j ,L,R,Z) );
	dyu[i][j] = dyd[i][j] + DELTA_TAU * dy ;
}

//===================================End Simulation===============

//=========================================================================

int cout_result ( int n, float ( *dp )[YLEN], const char * type ,int L, int R, int Z, char* filename2, float& evaluate)
{
	int index=0,Y_max=0,X_index=0;
	float min, max;
	int i, j;
	min = max = dp[L][0];
	for(i=L; i<=R; i++){
		for(j=0; j<=Z; j++){
			if(min>dp[i][j]) min = dp[i][j];
			if(max<dp[i][j]) max = dp[i][j];
		}
	}
	char file1[256],file2[256];

	sprintf ( file2, "%s/%s_%.3d.bmp",filename2,type,n);
	float tran_factor = ( max - min ) / 255;
	CImg < unsigned char > img ( XLEN, YLEN );
	for( i = 0; i < XLEN; ++i ) {
		for ( j = 0; j < YLEN; ++j )
		{
			img ( i, j ) = 0;
			unsigned char c = 0;
			if( i>=L && i<=R && j<=Z ){
				int b = ( dp[i][j] - min ) / tran_factor;
				if(b>=100)
				{
					img(i,j)=255;
					if(j>Y_max)
						Y_max=j;
```

```
                    }
                    else    img(i,j)=0;

                    c = (dp[i][j] - min ) / tran_factor;
                    if( c < 255 ) c++;
                }//end if
            }
        }

        for(j=0;j<YLEN;j++)
        {
            if(img(10,j)==255||img(190,j)==255)
            {
                X_index=1;
                break;
            }
        }
        for(i=60;i>0;i--)
        {
            if(img(i,3)==255)
            {
                X_index=1;
                break;
            }
        }

        if(Y_max>=180||X_index||n==350)
        {
            index=1 ;    img.save_bmp ( file2 );
        }
        if(index){
            //========================skeleton extraction=========================
            int k = 0,l = 0;
            for(k=0;k<YLEN;k++)
            {
                for(l=0;l<XLEN;l++)
                {
                    if(img(l,k)==255)    img(l,k) = 1;
                    else    img(l,k) = 0;
                }
            }
            CImg < unsigned char > dst( XLEN, YLEN );
            for(i=0;i<YLEN;i++)
            {
```

```
        for(j=0;j<XLEN;j++)
        {
            dst(j,i)=img(j,i) ;
        }
    }

    CImg < unsigned char > t_image( XLEN, YLEN );
    i = 0;j = 0;
    for(int G_n=0; G_n<30; G_n++)
    {
        for(i=0;i<YLEN;i++)
        {
            for(j=0;j<XLEN;j++)
            {
                t_image(j,i) =dst(j,i);
            }
        }
        for(i=0;i<YLEN;i++)
        {
            for(j=0; j<XLEN; j++)
            {
                if(t_image(j,i)==1)
                {
                    int ap=0;
                    int p2 = (i==0)?0:t_image(    j,i-1);
                    int p3 = (i==0 || j==XLEN-1)?0:t_image( j+1,i-1);
                    if (p2==0 && p3==1) ap++;
                    int p4 = (j==XLEN-1)?0:t_image(j+1,i);
                    if(p3==0 && p4==1)    ap++;
                    int p5 = (i==YLEN-1 || j==XLEN-1)?0:t_image( j+1,i+1);
                    if(p4==0 && p5==1)    ap++;
                    int p6 = (i==YLEN-1)?0:t_image(j,i+1);
                    if(p5==0 && p6==1)    ap++;
                    int p7 = (i==YLEN-1 || j==0)?0:t_image(j-1,i+1);
                    if(p6==0 && p7==1)    ap++;
                    int p8 = (j==0)?0:t_image(j-1,i);
                    if(p7==0 && p8==1)    ap++;
                    int p9 = (i==0 || j==0)?0:t_image(j-1,i-1);
                    if(p8==0 && p9==1)    ap++;
                    if(p9==0 && p2==1)    ap++;
                    if((p2+p3+p4+p5+p6+p7+p8+p9)>1                                  &&
(p2+p3+p4+p5+p6+p7+p8+p9)<7)
                    {
                        if(ap==1)
```

```
                    {
                        if(!(p2 && p4 && p6))
                        {
                            if(!(p4 && p6 && p8)) dst(j,i)=0;
                        }
                    }
                }
            }//end if
        }
    }
    for(i=0;i<YLEN;i++)
    {
        for(j=0;j<XLEN;j++)
        {
            t_image(j,i) =dst(j,i);
        }
    }
    for(i=0; i<YLEN;i++)
    {
        for(int j=0; j<XLEN; j++)
        {
            if(t_image(j,i)==1)
            {
                int ap=0;
                int p2 = (i==0)?0:t_image(j,i-1);
                int p3 = (i==0 || j==XLEN-1)?0:t_image(j+1,i-1);
                if (p2==0 && p3==1) ap++;
                int p4 = (j==XLEN-1)?0:t_image(j+1,i);
                if(p3==0 && p4==1)    ap++;
                int p5 = (i==YLEN-1 || j==XLEN-1)?0:t_image(j+1,i+1);
                if(p4==0 && p5==1)    ap++;
                int p6 = (i==YLEN-1)?0:t_image(j,i+1);
                if(p5==0 && p6==1)    ap++;
                int p7 = (i==YLEN-1 || j==0)?0:t_image(j-1,i+1);
                if(p6==0 && p7==1)    ap++;
                int p8 = (j==0)?0:t_image(j-1,i);
                if(p7==0 && p8==1)    ap++;
                int p9 = (i==0 || j==0)?0:t_image(j-1,i-1);
                if(p8==0 && p9==1)    ap++;
                if(p9==0 && p2==1)    ap++;
                if((p2+p3+p4+p5+p6+p7+p8+p9)>1                                  &&
(p2+p3+p4+p5+p6+p7+p8+p9)<7)
                {
                    if(ap==1)
```

```
                    {
                        if(p2*p4*p8==0)
                        {
                            if(p2*p6*p8==0) dst(j,i)=0;
                        }
                    }
                }
            }
        }
    }
}
i = 0;j = 0;
for(i=0;i<XLEN;i++)
{
    for(j=0;j<YLEN;j++)
    {
        if(dst(i,j)==1)    dst(i,j) = 255;
        else    dst(i,j) = 0;
        if(img(i,j)==1)    img(i,j) = 255;
        else    img(i,j) = 0;
    }
}
//==========================End skeleton extraction=====================

//=======================feature extraction==========================
feature_mode=0;
feature_width=0;
feature_d=0;
feature_length=0;
feature_cebranch=0;
feature_branch=0;

int L_feature_d[10]={0,0,0,0,0,0,0,0,0,0};
int R_feature_d[10]={0,0,0,0,0,0,0,0,0,0};

int width_count,width_min=100;
int    L_num=0;

int L_dis=0,L_i=0;
int R_dis=0,R_i=0;

for(int k=5;k<YLEN;k++)
{
    width_count=0;
```

```
int row=k,col=XLEN/2;

int L_idx=dst(XLEN/2,k)+dst(XLEN/2-1,k) +dst(XLEN/2-2,k);
int R_idx=dst(XLEN/2,k)+dst(XLEN/2+1,k) +dst(XLEN/2+2,k);

int M_idx=dst(XLEN/2,k+5)+dst(XLEN/2-1,k+5) +dst(XLEN/2-2,k+5)+dst(XLEN/2+1,k+5) +dst(XLEN/2+2,k+5);
if( L_idx>=255*2&&(k-L_dis!=1||L_dis==0)&&L_i<10)
{
    if(M_idx){
        feature_mode=1;
        L_feature_d[L_i++]=k-L_dis;
        L_dis=k;
    }
    else //(!M_idx)
    {
        if(L_i-1<2) { feature_mode=3;}
        else feature_mode=4;
        int T_index=0;
        if(dst(XLEN/2-1,k)==255)
        {
            row=k; col=XLEN/2-1;
            L_num++;
            T_index=1;
            while(T_index)
            {
                T_index=0;
                if(dst(col-1,row-1)==255)
                {
                    int row_idx=row;    int col_idx=col;
                    float slope1=((float)(row_idx-k)/(float)(XLEN/2-col_idx));
                    FindBranch(dst,row_idx,col_idx);
                    float slope2=((float)(row_idx-row)/(float)(col-col_idx+0.0000001));
                    if(abs(slope1-slope2)<0.6)    {    feature_mode=4; feature_branch=1;}
                    else    {
                        if(feature_branch) break;
                        else {Down_keepgoing(dst,row,col);
                        Up_keepgoing(dst,row,col);
                        break;}
                    }
                }
                else if(dst(col-1,row+1)==255)
```

```
{
    if(dst(col,row+1)+dst(col,row+2)==255*2)
    {
        int row_idx=row;    int col_idx=col;
        float        slope1=((float)(row_idx-k)/(float)(XLEN/2-
col_idx));
        FindBranch(dst,row_idx,col_idx);
        float          slope2=((float)(row_idx-row)/(float)(col-
col_idx+0.0000001));
        if(abs(slope1-slope2)<0.6)       {   feature_mode=4;
feature_branch=1;}
        else {
            if(feature_branch) break;
            else {Down_keepgoing(dst,row,col);
            Up_keepgoing(dst,row,col);
            break;}
        }
    }
    if(dst(col-2,row+1)+dst(col-
1,row)==255*2&&(dst(col,row+1)==255||dst(col,row+2)))
    {
        int row_idx=row;    int col_idx=col;
        float        slope1=((float)(row_idx-k)/(float)(XLEN/2-
col_idx));
        FindBranch(dst,row_idx,col_idx);
        float          slope2=((float)(row_idx-row)/(float)(col-
col_idx+0.0000001));
        if(abs(slope1-slope2)<0.6)           {feature_mode=4;
feature_branch=1;}
        else   {
            if(feature_branch) break;
            else {Down_keepgoing(dst,row,col);
            Up_keepgoing(dst,row,col);
            break;}
        }
    }

  if(dst(col,row+1)==255&&(dst(col+1,row+1)==255||dst(col+1,row+2)))
    {
        int row_idx=row;    int col_idx=col;
        float        slope1=((float)(row_idx-k)/(float)(XLEN/2-
col_idx));
        FindBranch(dst,row_idx,col_idx);
        float          slope2=((float)(row_idx-row)/(float)(col-
```

```
col_idx+0.0000001));
                        if(abs(slope1-slope2)<0.6)        {feature_mode=4;
feature_branch=1;}
                        else {
                            if(feature_branch) break;
                            else {Down_keepgoing(dst,row,col);
                            Up_keepgoing(dst,row,col);
                            break;}
                        }
                    }
                    if(dst(col-1,row)==255&&dst(col-2,row-1)==255)
                    {
                        int row_idx=row;    int col_idx=col;
                        float        slope1=((float)(row_idx-k)/(float)(XLEN/2-
col_idx));
                        FindBranch(dst,row_idx,col_idx);
                        float          slope2=((float)(row_idx-row)/(float)(col-
col_idx+0.000001));
                        if(abs(slope1-slope2)<0.6)      {   feature_mode=4;
feature_branch=1;}
                        else   {
                            if(feature_branch) break;
                            else {Down_keepgoing(dst,row,col);
                            Up_keepgoing(dst,row,col);
                            break;}
                        }
                    }
                    L_num++;
                    T_index=1;
                    row=row+1; col=col-1;
                    continue;
                }
                else if(dst(col-1,row)==255)
                {
                    if(dst(col,row+1)==255) break;
                    L_num++;
                    T_index=1;
                    row=row; col=col-1;
                    continue;
                }
                else if(dst(col,row+1)==255)
                {
                    if(dst(col-
1,row+2)==255&&dst(col,row+2)==255&&(dst(col+1,row+3)==255||dst(col+1,row+2)==255))
```

```
                    {feature_mode=4; feature_branch=1;}
                    else if(dst(col,row+2)+dst(col,row+4)==255*2)
                    {   if(feature_branch)    break;
                    else {Down_keepgoing(dst,row,col);
                    Up_keepgoing(dst,row,col);
                    break;}
                    }
                    L_num++;
                    T_index=1;
                    row=row+1; col=col;
                    continue;
                }
            }//end while
        }//end
    }//end

    if(feature_mode==4||feature_mode==3)
    {

        width_min=100;
        if(k-10>33)
        {
            for(int i=33;i<k-10;i++){
                width_count=1;
                col=XLEN/2+1;
                while(img(col++,i)==255)
                {
                    width_count++;
                }
                if(width_count<width_min)    width_min=width_count;
            }
        }
        else
        {
            for(int i=k-15;i<k-10&&i>0;i++){
                width_count=1;
                col=XLEN/2+1;
                while(img(col++,i)==255)
                {
                    width_count++;
                }
                if(width_count<width_min)    width_min=width_count;
            }
        }
```

```
                break;
            }
            else
            {
                for(int i=k+15;i<k+25;i++)
                {
                    width_count=1;
                    col=XLEN/2+1;
                    while(img(col++,i)==255)
                    {
                        width_count++;
                    }
                    if(width_count<width_min)    width_min=width_count;
                }
            }
        }

        if(R_idx>=255*2&&(k-R_dis!=1||R_dis==0)&&R_i<10)
        {
            R_feature_d[R_i++]=k-R_dis;
            R_dis=k;
        }
    }

    if(feature_mode==4&&L_feature_d[0]){ feature_cebranch=1;}

    if(feature_mode==1)
    {
        int k=0;
        while(L_feature_d[k]&&R_feature_d[k])
        {
            if(abs(L_feature_d[k]-R_feature_d[k])<2) feature_mode=2;
            else {feature_mode=1;break;}
            k++;
        }
    }

    if(feature_mode==1||feature_mode==2){
        k=1;
        int sum=0;
        int Ld_max=0,Ld_min=200;
        int Rd_max=0,Rd_min=200;
        while(L_feature_d[k])
        {
```

```
			if(L_feature_d[k]>Ld_max) Ld_max=L_feature_d[k];
			if(L_feature_d[k]<Ld_min)    Ld_min=L_feature_d[k];
			sum+=L_feature_d[k];
			k++;
		}
		int s=1;
		while(R_feature_d[s])
		{
			if(R_feature_d[s]>Rd_max)    Rd_max=R_feature_d[s];
			if(R_feature_d[s]<Rd_min)    Rd_min=R_feature_d[s];
			sum+=R_feature_d[s];
			s++;
		}
		if(s+k>5){
			if(Rd_max>Ld_max) {sum-=Rd_max;}
			else sum-=Ld_max;
			if(Rd_min>Ld_min) sum-=Ld_min;
			else sum-=Rd_min;
			feature_d=sum/(s-1+k-1-2);
		}
		else{
			if(s==1&&k==1)
				feature_d=sum;
			else    feature_d=sum/(s-1+k-1);
		}
	}

	printf("\n----------------------------------------\n");
	switch(feature_mode){
	case 0: {
		printf("mode=%d\n",feature_mode);
		evaluate=1000000;
		break;
			}
	case 1: {
		printf("mode=%d\n",feature_mode);
		feature_width=width_min*2;
		printf("width:%d\n",feature_width);
		printf("d:%d\n",feature_d);
		if(feature_mode==C_feature_mode)
			evaluate=(C_feature_d-feature_d)*(C_feature_d-feature_d);
		else evaluate=500000;
		break;
			}
```

```
		case 2: {
			printf("mode=%d\n",feature_mode);
			feature_width=width_min*2;
			printf("width:%d\n",feature_width);
			printf("d:%d\n",feature_d);
			if(feature_mode==C_feature_mode)				evaluate=(C_feature_d-
feature_d)*(C_feature_d-feature_d);
			else evaluate=500000;
			break;
				}
		case 3: {
			printf("mode=%d\n",feature_mode);
			feature_width=width_min*2;
			printf("width:%d\n",feature_width);
			feature_length=L_num;
			printf("length:%d\n",feature_length);
			if(feature_mode==C_feature_mode)
			{
				if(1)
				{
					evaluate = (C_feature_length-feature_length)
						*(C_feature_length-feature_length);
				}
				else
					evaluate = 5000
					+(C_feature_length-feature_length)
					*(C_feature_length-feature_length);
			}
			else	evaluate=500000;
			break;
				}
		case 4: {
			printf("mode=%d\n",feature_mode);
			feature_width=width_min*2;
			printf("width:%d\n",feature_width);
			feature_length=L_num;
			printf("length:%d\n",feature_length);
			printf("cebranch=%d\n",feature_cebranch);
			printf("branch=%d\n",feature_branch);
			if(feature_mode == C_feature_mode)
			{
				if	(feature_branch	==	C_feature_branch	&&	feature_cebranch	==
C_feature_cebranch)
					evaluate	=	(C_feature_length-feature_length)*(C_feature_length-
```

```
feature_length);
                        else
                              evaluate=100000;
                  }

                  else { if(C_feature_mode==3) evaluate=300000;
                  else evaluate=500000;
                  }
                  break;
                        }
            }
            //=======End feature extraction============================================
      }
      return index;
}

//====================Cost function==================================
float evaluate(float* vector, int gen, int Num)
{
      float evaluate=0.0f;

      char bmpname[20];
      RHO_H=vector[1];
      C_O=vector[0];
      EPSILON=vector[2];
      MU=vector[3];
      D_H=vector[4];
      int biaoji=0;

      sprintf (bmpname, "bmps%d", gen);
      mkdir(bmpname,S_IRWXU | S_IRWXG | S_IROTH | S_IXOTH);
      /*------------------------------------------------------------------*/

      cudaSetDevice( 1 );
      int memsize = XLEN * YLEN * sizeof ( float ) ;

      float ( * a )[YLEN] = fa;
      float ( * h )[YLEN] = fh;
      float ( * s )[YLEN] = fs;
      float ( * y )[YLEN] = fy;

      float ( * dad )[YLEN];        cudaMalloc ( ( void ** )&dad, memsize );
      float ( * dau )[YLEN];        cudaMalloc ( ( void ** )&dau, memsize );
      float ( * dhd )[YLEN];        cudaMalloc ( ( void ** )&dhd, memsize );
```

```
	float ( * dhu )[YLEN];		cudaMalloc ( ( void ** )&dhu, memsize );
	float ( * dsd )[YLEN];		cudaMalloc ( ( void ** )&dsd, memsize );
	float ( * dsu )[YLEN];		cudaMalloc ( ( void ** )&dsu, memsize );
	float ( * dyd )[YLEN];		cudaMalloc ( ( void ** )&dyd, memsize );
	float ( * dyu )[YLEN];		cudaMalloc ( ( void ** )&dyu, memsize );

	int i,j;
	for(i=0; i<XLEN; i++){
		for(j=0; j<YLEN; j++){
			a[i][j] = h[i][j] = y[i][j] =0.00001f;
			s[i][j] = 1.0f;
			if(i<=XLEN/2+1 && i>=XLEN/2-1 && j>=12 && j<=12+8){
				a[i][j] = 2.0f; h[i][j] = 0.002f; y[i][j] = 1.0f;
			}
		}
	}
	cudaMemcpy	( dad, a, memsize, cudaMemcpyHostToDevice );
	cudaMemcpy	( dhd, h, memsize, cudaMemcpyHostToDevice );
	cudaMemcpy	( dsd, s, memsize, cudaMemcpyHostToDevice );
	cudaMemcpy	( dyd, y, memsize, cudaMemcpyHostToDevice );
	cudaMemcpy	( dau, a, memsize, cudaMemcpyHostToDevice );
	cudaMemcpy	( dhu, h, memsize, cudaMemcpyHostToDevice );
	cudaMemcpy	( dsu, s, memsize, cudaMemcpyHostToDevice );
	cudaMemcpy	( dyu, y, memsize, cudaMemcpyHostToDevice );
#define BLOCK_SIZE_X	2
#define BLOCK_SIZE_Y	32
	dim3 dimBlock ( 2, 32 );
	dim3 dimGrid	( ( XLEN + BLOCK_SIZE_X - 1 ) / BLOCK_SIZE_X, ( YLEN + BLOCK_SIZE_Y - 1 ) /
BLOCK_SIZE_Y );

#define X0			10
#define Y0			20

	int L = 0, R = XLEN-1, Z = YLEN-1;
	for ( int t = 0; t <= TAU; ++t ){
		update <<< dimGrid, dimBlock >>> ( dad, dau, dhd, dhu, dsd, dsu, dyd,
dyu,L,R,Z,t,C_O,RHO_H,EPSILON,MU,D_H);
		float ( * tmp )[YLEN];
		tmp = dad; dad = dau; dau = tmp;
		tmp = dhd; dhd = dhu; dhu = tmp;
		tmp = dsd; dsd = dsu; dsu = tmp;
		tmp = dyd; dyd = dyu; dyu = tmp;

		if ( t % TSTEP ) continue;
```

```
#define rho_x                   1
#define rho_y                   2*rho_x

            if ( 0 )
            {
                  L -= rho_x;
                  R += rho_x;
                  Z += rho_y;
                  L = (L>=0)?L:0;    R = (R<XLEN)?R:XLEN-1; Z = (Z<YLEN)?Z:YLEN-1;
            }
            cudaMemcpy    ( y, dyd, XLEN * YLEN * sizeof ( float ), cudaMemcpyDeviceToHost );
            char type[20];
            sprintf (type, "y%d", Num);
            biaoji=cout_result ( t/TSTEP, y, type ,L,R,Z,bmpname,evaluate);
            if(biaoji||t/TSTEP==350)    { printf("\nk=%f\n",evaluate); break; }
      }
      if(evaluate<10000)
      {
            FILE* f = fopen("3_15_6.txt","a+");
            fprintf(f,"----------------------------\n");
            fprintf(f,"mode=%d\n",feature_mode);
            fprintf(f,"width:%d\n",feature_width);
            fprintf(f,"length:%d\n",feature_d);
            fprintf(f,"length:%d\n",feature_length);
            fprintf(f,"evaluate:%f\n",evaluate);
            fprintf(f,"%f**%f**%f**%f**%f\n",C_O,RHO_H,EPSILON, MU, D_H);
            fprintf(f,"count=%d**NP=%d**\n",gen,Num);
            fprintf(f,"------------------------------\n");
            fclose(f);
      }
      cudaFree ( dad      );
      cudaFree ( dau      );
      cudaFree ( dhd      );
      cudaFree ( dhu      );
      cudaFree ( dsd      );
      cudaFree ( dsu      );
      cudaFree ( dyd      );
      cudaFree ( dyu      );

      return evaluate;
}

//===========DE Algorithm==============================================
```

```
#define    gen_max       10
#define    NP            30
#define    DE_D          5
#define    CR            0.3f
#define    DE_F          0.6f

//=================================================================
int main( int argc, char const *argv[] )
{
        float target[NP][DE_D],mutant[NP][DE_D],trial[DE_D];
        int count=1;
        int r1,r2,r3,q,w;
        float cost[NP],score=1;
        bool      DE_idx=0;
        float mid[DE_D];
        FILE *f = fopen("para0.txt","w");
        for(q=0;q<NP;q++)
        {
                srand48 ( time ( NULL ) );
                target[q][0]=drand48()*0.1f+0.01f;                              //c0=[0.01,0.1]
                target[q][1]=drand48()*0.00015f+0.00005f;               //RHO_H=[0.00005,0.00015]
                target[q][2]=drand48()*1.5f+0.05f;                          //EPSILON=[0.05,1.5]
                target[q][3]=drand48()*0.2f+0.1f;                   //mu=[0.1,0.2]
                target[q][4]=rdrand48()*0.3f+0.2f;                          //D_h=[0.2,0.3]


                for(w=0;w<DE_D;w++)
                        mid[w]=target[q][w];
                cost[q]=evaluate(mid,0,q);


        fprintf(f,"%f %f %f %f %f\n",target[q][0],target[q][1],target[q][2],target[q][3],target[q][4]);
                if(cost[q]<10000) printf("======count=0****DE_D=%d=========\n",q);

        }
        fclose(f);
        int i,j;
        while(count<gen_max && DE_idx==0)
        {
                char filename[20];
                sprintf(filename, "para%d.txt", count);
                f = fopen(filename,"w");
                for(i=0;i<NP;i++)
                {
```

```
            do r1=rand()%NP; while(r1==i);                          //Randomly pick 3 indexes,
            do r2=rand()%NP; while(r2==i||r1==r2);              //integer,mutually different
            do r3=rand()%NP; while(r3==i||r2==r3||r1==r3);//and all different from i
            j=rand()%DE_D;
            for(int k=1;k<=DE_D;k++)
            {
                if((rand()%100)*0.01f<CR||k==DE_D) trial[j]=target[r3][j]+DE_F*(target[r1][j]-
target[r2][j]);
                else    trial[j]=target[i][j];
                j=(j+1)%DE_D;
            }
            fprintf(f,"%f %f %f %f %f\n",trial[0],trial[1],trial[2],trial[3],trial[4]);
            score=evaluate(trial,count,i);
            printf("\n--------------------------------------------\n");

            if(score<10000) printf("count=%d**NP=%d**\n",count,i);

            if(score<0.0001)
            {
                DE_idx=1;
                break;
            }
            /*------------------------------------------------------------------------*/
            if(score<=cost[i])
            {
                for(j=0;j<DE_D;j++)    mutant[i][j]=trial[j];
                cost[i]=score;
            }
            else     for(j=0;j<DE_D;j++)    mutant[i][j]=target[i][j];
        }   //end for
        fclose(f);
        if(DE_idx) {printf("\n----Oops----\n");break;}

        for(i=0;i<NP;i++)
        {
            for(j=0;j<DE_D;j++)
                target[i][j]=mutant[i][j];
        }
        count++;
    }//End while.

    printf("\n----------------------------------------\n");
    for(i=0;i<NP/5;i++)
printf("\n%f    %f    %f    %f    %f\n",cost[i*5],cost[i*5+1],cost[i*5+2],cost[i*5+3],cost[i*5+4]);
```

```
	printf("\ncount=%d\n",count);

	if(!DE_idx)
	{
		float cost_min=cost[0];
		int minNum=0;
		for(i=1;i<NP;i++)
		{
			if(cost[i]<cost_min)
			{
				cost_min=cost[i];
				minNum=i;
			}
		}
		for(j=0;j<DE_D;j++)
			trial[j]=target[minNum][j];
	}
	printf("\n--------------Output----------------------\n");
	printf("%f**%f**%f**%f**%f\n",trial[0],trial[1],trial[2],trial[3],trial[4]);

	return 0;
}
```
