# Supplementary material for: Automated numerical simulation of biological pattern formation based on visual feedback simulation framework
Source: PLoS One. 2017 Feb 22;12(2):e0172643. doi: 10.1371/journal.pone.0172643 (PMC5321435; doi:10.1371/journal.pone.0172643)
Supplement: S1 Text — The quantitative pattern features of VMCs, the pattern topologies and quantitative pattern features of lung branching are extracted by image processing automatically. We show the image processing method in the S1 Text. (DOCX) [file pone.0172643.s003.docx]

##### Quantitative pattern feature extractions of VMCs

The quantitative pattern features of VMCs include the pattern-area-to-total-area ratio $R^{1}$ and the perimeter-area ratio of the pattern $R^{2}$. The simulation images are processed by image binarization and edge detection before extract these features[1]. The threshold value in image binarization is set to a fixed value 180, due to the strong contrast and the low noise of the simulation images. $R^{1}$ and $R^{2}$ are calculated based on the binary images and the edge images by the following equations.

$$R^{1}=\frac{number of black pixels in binary image}{length*width of pattern image} (1)$$

$$R^{2}=\frac{number of edge pixels (white pixel) in edge image}{number of black pixels in binary image} (2)$$

Fig 1 shows the image processing results of three types of the VMCs simulation patterns. Then the quantitative pattern features $R^{1}$ and $R^{2}$ are calculated, which are shown in Table 1.

| A | 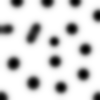 | B | 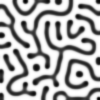 | C | 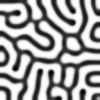 |
| --- | --- | --- | --- | --- | --- |
| D | 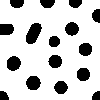 | E | 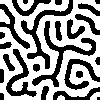 | F | 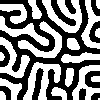 |
| G | 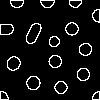 | H | 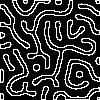 | I | 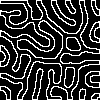 |

**Fig 1 Image processing results of the VMCs simulation patterns.** The first line (A-C) shows three types of the VMCs simulation pattern images, including spots (A), stripe (B) and labyrinthine (stripe doubling) (C). The second line (D-F) shows the binary images of the simulation images. The third line (G-I) shows the edge images of the simulation images, the edges are detected in binary pattern images.

Table 1 The quantitative pattern features of the VMCs simulation patterns

| Pattern | $R^{1}$ | $R^{2}$ |
| --- | --- | --- |
| Spot pattern | 0.20 | 0.37 |
| Stripe pattern | 0.41 | 0.54 |
| Labyrinthine pattern | 0.49 | 0.44 |

##### Pattern topology and pattern feature extractions of lung branching

We extract the pattern topology and the pattern features of lung branching based on the skeleton of the branching. A fast parallel algorithm for thinning digital patterns is utilized to extract the skeleton [2]. Fig 2 shows four types of the simulation branching pattern images and the corresponding skeleton images.

| A | 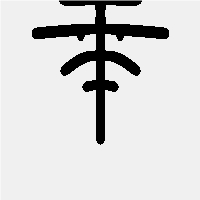 | B | 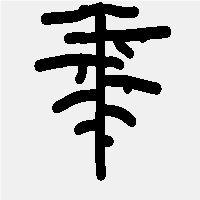 | C | 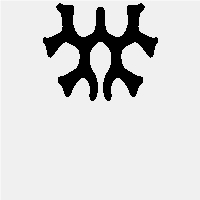 | D | 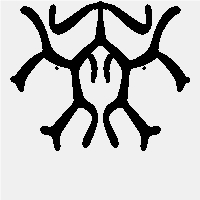 |
| --- | --- | --- | --- | --- | --- | --- | --- |
| E | 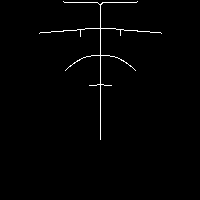 | F | 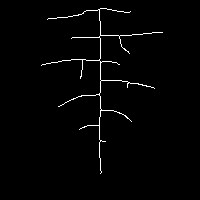 | G | 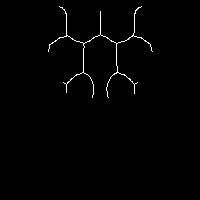 | H | 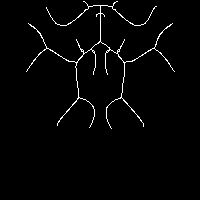 |

**Fig 2 Image skeleton extraction results of the simulation branching pattern images.** The first line (A-D) shows four types of the lung branching patterns in simulation, including zygomorphic side branching pattern (A), alternating side branching pattern (B), tip bifurcation pattern (C) and hybrid branching pattern (D). The second line (E-H) shows the corresponding skeleton images of the lung branching patterns.

First, the pattern topologies are obtained by pixel scanning in the skeleton images. We judge whether there is a long main stalk in the unknown branching pattern. If the main stalk is monopodial branching, we further confirm that the unknown pattern belongs to the zygomorphic side branching pattern or alternating side branching pattern based on the symmetry of the side branching. If the main stalk bifurcates into two equal-sized branches, we further judge whether the unknown pattern has side branches or not. If so, then the pattern belongs to the hybrid branching pattern; otherwise, the pattern belongs to the tip branching pattern.

Second, the branch length $d$, which is set to a quantitative pattern feature, is extracted by counting the number of pixels in the skeleton images. For the zygomorphic side branching pattern and alternating side branching pattern, we find the intersection pixel of the main stalk and side branches, and we then count the number of pixels between two adjacent intersection pixels from the side branches on the same side as the branch length. For the tip bifurcation pattern and hybrid branching pattern, we scan the pixels from one bifurcation pixel along the growth direction to the next bifurcation pixel, and we then count the number of pixels between two bifurcation pixels as the branch length. Fig 3 shows the process of the pattern topology and pattern feature extractions.

**Fig 3 The flow chart of pattern topology and pattern feature extractions.**

[1] Sonka M, Hlavac V, Boyle R. Image processing, analysis, and machine vision. 3rd ed. Cengage Learning; 2007.

[2] Zhang T Y, Suen C Y. A fast parallel algorithm for thinning digital patterns. Communications of the ACM. 1984; 27(3): 236-239.
